# A Golgi apparatus-related signature for predicting prognosis and evaluating the tumor immune microenvironment of uveal melanoma

**DOI:** 10.1097/MD.0000000000042909

**Published:** 2025-06-20

**Authors:** Xian Ge, Fei Ge, Longqin Xiao, Anting Su

**Affiliations:** a Jianyang Eye Hospital of Jianhu County, Yan Chen, Jiangsu, China.

**Keywords:** consensus clustering, drug sensitivities, golgi apparatus, immune infiltration, uveal melanoma

## Abstract

Uveal melanoma (UVM), a highly invasive and metastatic primary eye cancer with poor prognosis, contributes significantly to melanoma-related deaths despite being less common. Despite advances in therapy, the mortality rate remains unchanged due to frequent liver metastases and limited effective prognostic biomarkers. This study employed gene expression data from The Cancer Genome Atlas and Gene Expression Omnibus databases to investigate Golgi apparatus-related gene sets (GGRGs) in UVM. Survival analysis, consensus clustering, and principal component analysis were conducted to identify GGRGs associated with patient outcomes. Additionally, tumor microenvironment was assessed using IOBR tools, and a nomogram was constructed based on Cox regression models for predicting survival probabilities. The biological function of carbohydrate sulfotransferase protein family (CHST9) was evaluated by colony formation assay, transwell invasion assay, and wound healing assay. Univariate Cox regression identified 343 GGRGs significantly correlated with UVM prognosis. Consensus clustering revealed 2 distinct subtypes (cluster1 and cluster2) differing significantly in survival, with cluster2 showing more favorable outcomes. Principal component analysis effectively separated these clusters, while Kaplan–Meier curves confirmed their survival disparity. Least Absolute Shrinkage and Selection Operator Cox regression analysis pinpointed a 5-GGRGs-based signature, termed GGRGs-derived index (GGI), composed of lunatic fringe, KDELR3, CHST9, ATP8B3, and ACAN. This GGI stratified UVM cases into High_GGI and Low_GGI groups across multiple datasets, with the Low_GGI group consistently demonstrating significantly improved survival rates compared to the High_GGI group. Notably, the Low_GGI and High_GGI groups exhibited marked differences in clinicopathological characteristics, drug sensitivities, and immune infiltration levels. Ultimately, GGI and age emerged as independent prognostic factors for UVM and were incorporated into a nomogram, which displayed outstanding performance in predicting patient prognosis. Depletion of CHST9 expression dramatically inhibited the proliferative capacity of UVM cells, concurrently suppressing their metastatic activity and invasive properties. GGRGs are promising predictors of UVM prognosis and may inform personalized treatment strategies, contributing to a deeper understanding of the molecular mechanisms driving this aggressive cancer.

## 1. Introduction

Uveal melanoma (UVM) constitutes a less frequently encountered yet highly aggressive variant of melanoma, predominantly affecting adults and being recognized as the most prevalent primary malignancy within the ocular realm. Despite its relative rarity, representing merely 5% of all melanoma cases, UVM significantly contributes to an alarming 13% of melanoma-related mortalities.^[1]^ This oncological entity is associated with diverse risk parameters such as age demographics, racial disparities, and iris pigmentation patterns.^[2,3]^ While initial therapeutic interventions, including radiation therapy, transpupillary thermotherapy, and photodynamic treatment, are often employed for managing primary UVM lesions, approximately half of the patients experience metastatic progression, primarily in the hepatic organ, resulting in a median survival period of only 1-year post-metastasis.^[4,5]^ Despite recent advancements in ocular-sparing therapeutic modalities and the identification of novel prognostic biomarkers like Histone Deacetylase-2,^[6]^ Ataxia Telangiectasia and Rad3 Related Protein,^[7]^ and Nuclear Factor kappa B signaling pathway constituents,^[8]^ the mortality rate linked to UVM has remained stubbornly unaltered. The pressing necessity to augment our comprehension of UVM’s molecular and cellular pathophysiology persists due to the persistent threat of lethal metastases within a year and the unchanging mortality trend over the past decade.^[9]^ This underscores the paramount importance of identifying novel prognostic biomarkers and devising personalized therapeutic approaches to enhance clinical outcomes and improve survival rates among UVM patients.

The Golgi apparatus (GA) constitutes a fundamental cellular organelle that plays an indispensable role in orchestrating cellular functions and regulation through the facilitation of protein and lipid synthesis, modification, and trafficking from the endoplasmic reticulum to various cellular compartments.^[10]^ Recent scientific inquiries have accentuated its profound association with numerous pathologies, particularly breast cancer, wherein it significantly impacts pivotal processes such as cell proliferation, metastatic spread, and chemoresistance.^[11,12]^ Functioning as a central processing hub for intracellular substances, the GA is essential for the biosynthesis, maturation, and proper localization of proteins^[13]^; perturbations in its functionality are increasingly recognized as contributing factors to breast cancer initiation and progression.^[14]^ Notably, specific GA-related genes have been found to exhibit aberrant expression patterns or mutations within the context of breast cancer, conferring potential prognostic utility.^[15]^ The complex interplay between the GA and breast cancer biology not only unravels novel mechanisms underlying disease etiology but also presents opportunities to identify innovative therapeutic targets. Furthermore, the GA’s intricate crosstalk with other organelles like the endoplasmic reticulum, mitochondria, autophagosomes, and endosomes via membranous networks highlights its integral participation in cellular signaling cascades and effector responses during carcinogenesis.^[16,17]^ A comprehensive investigation into the functional dynamics of the GA and its associated gene repertoire holds paramount importance in refining patient prognosis and guiding the development of targeted therapeutic strategies.

In the present study, we have employed gene expression matrices obtained from The Cancer Genome Atlas (TCGA, accessible at https://portal.gdc.cancer.gov/) and the Gene Expression Omnibus (GEO, retrievable via http://www.ncbi.nlm.nih.gov/geo) databases to systematically investigate the expression patterns, mutational landscapes, and prognostic relevance of GGRGs in UVM. Subsequently, we have constructed GGRGs-based subtypes and a GGRGs-derived index (GGI), followed by extensive functional annotation, correlation analyses with clinical characteristics, assessments of immune infiltration, as well as evaluations of drug sensitivity. Our research findings have provided unique insights into the pivotal roles that GGRGs play in the progression and prognosis of UVM, thereby elucidating whether GGRGs could serve as viable prognostic biomarkers and potential therapeutic targets for patients diagnosed with UVM. These results not only deepen our understanding of the molecular underpinnings of UVM but also offer promising avenues for personalized treatment strategies informed by GGRGs profiles.

## 2. Materials and methods

### 2.1. Data acquisition

We obtained gene expression data from 80 UVM patients in the TCGA-UVM cohort, along with their corresponding clinical and pathological attributes, from the official Genomic Data Commons portal of TCGA project (https://portal.gdc.cancer.gov/). To uphold data quality and curtail possible bias, strict criteria were set for patient exclusion. Individuals were disqualified from the study if they satisfied any of these conditions: incomplete clinical information (e.g., missing age, pathological T stage, clinical stage, and gender); the follow-up duration was <30 days. Extremely brief follow-up intervals are likely to capture short-term postoperative problems or deaths unrelated to cancer, instead of results specific to the disease. Furthermore, we have retrieved transcriptomic and clinical information datasets from 2 cohorts in the GEO database, specifically GSE84976 and GSE22138, which encompass 28 and 63 unique cases of UVM, respectively.

### 2.2. Consensus clustering analysis

The prognostic relevance of GGRGs with respect to UVM was assessed through univariate Cox regression analysis, and those GGRGs demonstrating a statistically significant association (*P* < .05) were selected for further investigation via consensus clustering analysis. This analytical process was executed using the ConsensusClusterPlus package,^[18]^ employing the partitioning around medoids clustering algorithm and the “pearson” distance metric. Subsequently, survival analyses between derived subtypes were conducted using the survival and survminer packages, followed by principal component analysis to evaluate the molecular heterogeneity among the identified subtypes. Additionally, statistical comparisons of clinical and pathological characteristics across the different subtypes were performed to gain insights into their distinctive features.

### 2.3. Construction and evaluation of risk signature

Utilizing the glmnet package for the application of Least Absolute Shrinkage and Selection Operator (LASSO) Cox regression analysis,^[19]^ we advanced in compressing and selecting GGRGs that are significantly associated with prognosis. Subsequently, a GGI was computed using the following formula: GGI = ∑(βi × expi), where βi represents the coefficient attributed to gene i within the model, and expi signifies the expression level of gene i. Upon calculating the GGI, patients across the TCGA-UVM, GSE84976, and GSE22138 cohorts were dichotomized into 2 groups, designated as High_GGI and Low_GGI, based on their respective median GGI values. This stratification was followed by the execution of Kaplan–Meier survival analysis and principal component analysis to comprehensively evaluate the risk profiles of these groups.

### 2.4. Gene Ontology and Kyoto Encyclopedia of Genes and Genomes (KEGG) enrichment analysis

Initially, differential expression analysis was conducted using the edgeR package to identify genes differentially expressed between the High_GGI and Low_GGI groups with a significance threshold set at *P* = .05 and an absolute log2 fold change >2.^[20]^ Subsequently, functional enrichment analyses were performed via the clusterProfiler package,^[21]^ encompassing biological processes, molecular functions, cellular components from Gene Ontology terms, as well as KEGG pathways. Significantly enriched terms (*P* < .05) were selected and visually represented according to their respective categories.

### 2.5. Drug sensitivity analysis

Drug sensitivity assessments for 45 drugs, including Axitinib, Bexarotene, Bicalutamide, Bleomycin, and Bortezomib, among others, were carried out for patients in the TCGA-UVM cohort using the pRRophetic package.^[22]^ Sensitivity predictions were made employing the pRRopheticPredict() function, where tissueType was specified as “all” and batch correction was conducted using the ComBat method. Ultimately, comparative analyses were conducted to discern differences in drug sensitivity profiles between the distinct groups.

### 2.6. Tumor microenvironment analysis

Employing the IOBR package,^[23]^ a comprehensive analysis of the tumor microenvironment was conducted, which incorporates multiple algorithms for assessing immune infiltration within tumors, such as CIBERSORT, EPIC, xCell, MCP-counter, ESTIMATE, TIMER, quanTIseq, and IPS. This analysis involved comparing differences in immune cell infiltration between different groups, along with evaluating Stromalscore, Immunescore, ESTIMATEscore, and Tumor purity. Furthermore, the correlation between GGI and IPS was examined.

### 2.7. Construction and evaluation of nomogram

Utilizing univariate and multivariate Cox regression analyses, we assessed the prognostic value of GGI in conjunction with clinical and pathological characteristics. Independent prognostic factors with *P* < .05 were identified and used to construct a nomogram for predicting the 1-, 2-, and 3-year overall survival in UVM patients using the rms package. The performance of this nomogram was evaluated through receiver operating characteristic curves, decision curve analysis, and calibration curves; the decision curve analysis was performed utilizing the rmda package.

### 2.8. Cell culture

Human UVM cell line OMM2.5 was cultured in RPMI-1640 medium supplemented with 10% fetal bovine serum and 1% penicillin-streptomycin at 37 °C under 5% CO_2_. Cells were harvested at 80% to 90% confluence for RNA and protein extraction.

### 2.9. RNA extraction and qRT-PCR

Total RNA will be extracted using TRIzol reagent. cDNA synthesis will be performed with reverse transcriptase. Primers specific for carbohydrate sulfotransferase protein family (CHST9) (Forward: 5’-AAGAAAAAGCACATGTGTTA-3’; Reverse: 5’-CAGATGGCTGCATTTCTCCT-3’) and reference gene GAPDH (Forward: 5’-CGGAGTCAACGGATTTGGTCGTAT-3’; Reverse: 5’-AGCCTTCTCCATGGTGGTGAAGAC-3’) will be designed using Primer-BLAST. SYBR Green-based qRT-PCR will quantify mRNA expression.

### 2.10. Western blotting

Total proteins were extracted from logarithmically growing cells using RIPA lysis buffer (Sigma–Aldrich). Protein lysates were separated by SDS-PAGE and transferred to PVDF membranes. After blocking with 5% nonfat milk, membranes were incubated with anti-CHST9 primary antibody (1:1000, CUSABIO) and HRP-conjugated secondary antibody (1:5000). Signals were detected using an ECL kit, with GAPDH as the loading control.

### 2.11. siRNA design and transfection

Three CHST9-specific siRNA sequences si-CHST9 and a scrambled siRNA negative control (si-NC) were synthesized (GenePharma). UVM cells were transfected with 50 nM siRNA using Lipofectamine 3000 reagent for 48 to 72 hours. Knockdown efficiency was validated by qRT-PCR and Western blotting.

### 2.12. Colony formation assay

Cells (500 cells/well) were cultured in 6-well plates for 10 to 14 days. Colonies were fixed with methanol, stained with 0.1% crystal violet, and counted using ImageJ software.

### 2.13. Wound healing and transwell invasion assays

For migration, a scratch wound was created in confluent monolayers, and wound closure was measured at 24 hours. For invasion, Matrigel-coated Transwell chambers were used, and invaded cells were stained and quantified.

### 2.14. Statistical analyses

All statistical analyses and visualizations were conducted using R software (version 4.3.2) and (GraphPad Prism 10.0). Comparisons between groups were analyzed using the Student *t* test, Wilcoxon test or one-way ANOVA, as appropriate. A *P*-value < .05 was considered statistically significant.

## 3. Results

### 3.1. GGRGs-based consensus clustering identifies 2 distinct subtypes of UVM

**Figure 1.**
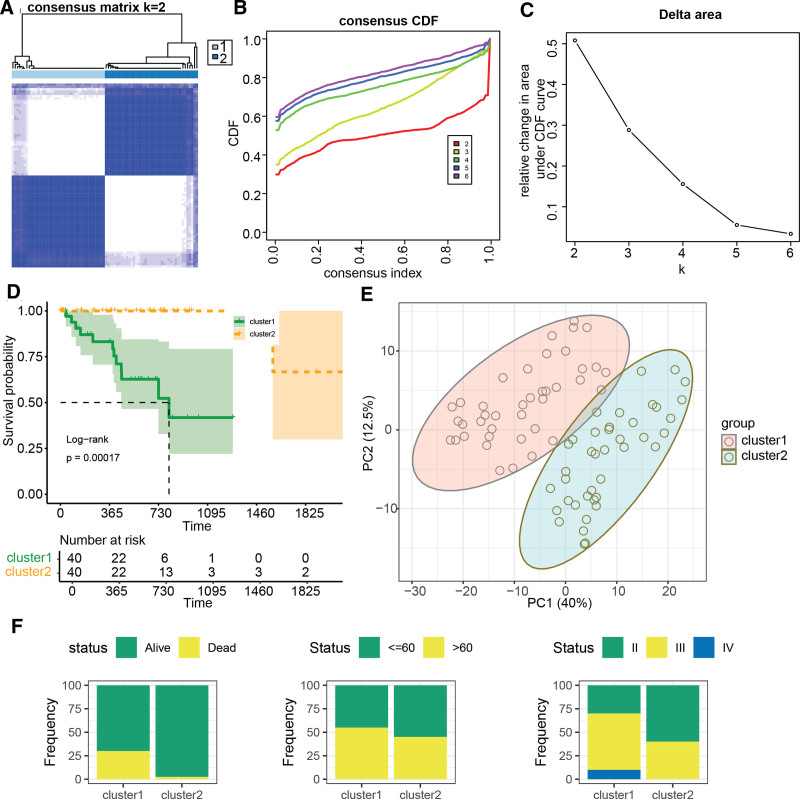
Consensus clustering analysis based on prognosis-related Golgi apparatus associated genes. (A) Consensus matrix heatmap (k = 2) illustrating the probability of 2 patients belonging to the same cluster across TCGA-UVM samples, as determined by prognosis-related GGRGs. (B) Consensus Cumulative Distribution Function (CDF) plot. (C) Delta area plot in consensus clustering, indicating that when the Delta area value stabilizes and the line in the plot flattens, the optimal number of clusters is deemed to be reached. (D) Kaplan–Meier (KM) survival curve analysis for clusters derived from GGRG-related classification. (E) Principal component analysis (PCA) plot based on prognosis-related GGRGs, demonstrating the separation between different clusters. (F) The distribution of status (alive/deceased), age, and stage among the identified clusters. GGI = GGRGs-derived index, GGRGs = Golgi apparatus-related gene sets, TCGA = The Cancer Genome Atlas, UVM = uveal melanoma.

Univariate Cox regression analysis revealed that out of 1643 GGRGs, 343 were significantly associated with the prognosis of UVM (Table S1, Supplemental Digital Content, https://links.lww.com/MD/P217). Based on a consensus clustering analysis using these prognosis-related GGRGs, the TCGA-UVM cohort was stratified into 2 distinct subtypes, designated as cluster1 and cluster2 (Fig. 1A–C). Survival analysis demonstrated that patients in cluster2 had significantly better outcomes compared to those in cluster1 (Fig. 1D, *P* = .00017). Principal component analysis based on the prognosis-associated GGRGs showed clear separation between cluster1 and cluster2 (Fig. 1E). Figure 1F illustrates that within the poorer prognosis cluster1, there is a higher proportion of deceased UVM patients, as well as a greater number of elderly patients and those with advanced-stage disease.

### 3.2. GGRGs-derived GGI represents a risk signature for UVM

**Figure 2.**
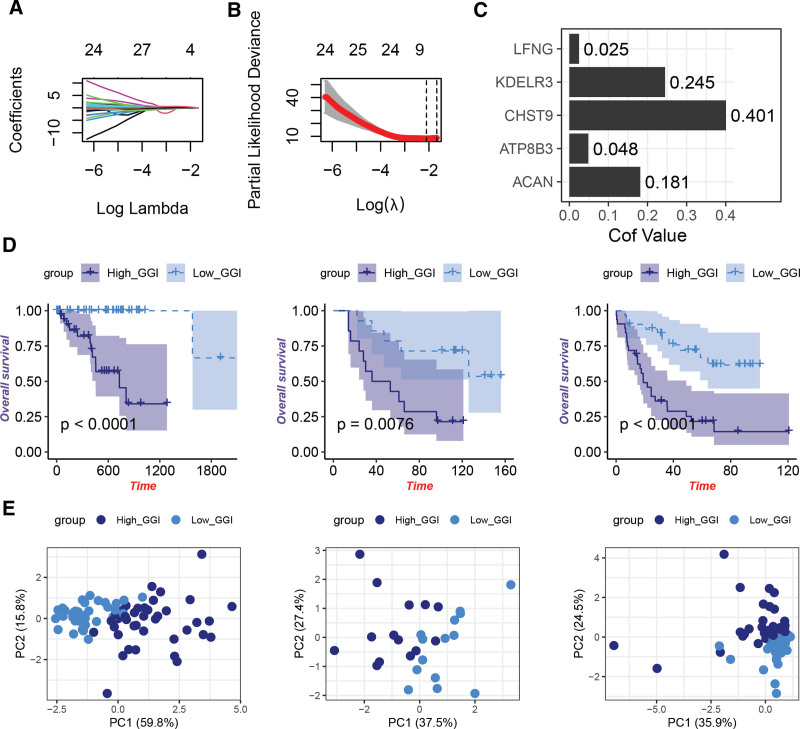
Construction of a GGRGs-derived index (GGI) for UVM based on prognosis-related GGRGs. (A, B) LASSO Cox regression optimization to select 5 GGRGs forming a risk signature for UVM. (C) Coefficients of the genes composing the GGI. (D) Survival analysis comparing High_GGI and Low_GGI groups in TCGA-UVM, GSE84976, and GSE22138 cohorts. (E) Principal component analysis (PCA) plot for TCGA-UVM, GSE84976, and GSE22138 cohorts based on 5 GGRGs. GGI = GGRGs-derived index, GGRGs = Golgi apparatus-related gene sets, LASSO = Least Absolute Shrinkage and Selection Operator, TCGA = The Cancer Genome Atlas, UVM = uveal melanoma.

A LASSO Cox analysis with penalty parameter tuning, established via tenfold cross-validation, was conducted to further refine the set of GGRGs. Ultimately, 5 signature-related genes were confirmed through LASSO regression analysis, exhibiting non-zero coefficients (Fig. 2A, B). The formula for the GGI score was derived as follows: GGI = 0.025 lunatic fringe (LFNG) + 0.245KDELR3 + 0.401CHST9 + 0.048ATP8B3 + 0.181*ACAN. According to the median value of GGI, patients in the TCGA-UVM, GSE84976, and GSE22138 cohorts were stratified into High_GGI and Low_GGI groups, where the Low_GGI group displayed significantly better prognosis than the High_GGI group (Fig. 2D). Principal component analysis based on the genes composing the GGI score demonstrated clear separation between the High_GGI and Low_GGI groups (Fig. 2E).

### 3.3. Association of GGI with UVM clinical pathological characteristics and somatic mutations

**Figure 3.**
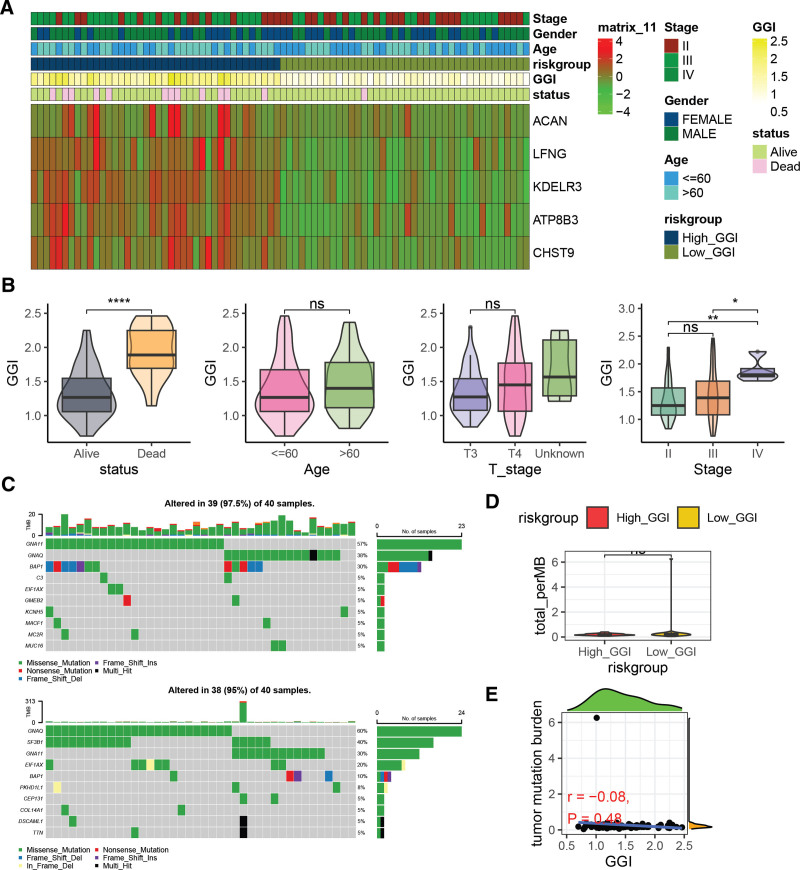
Clinical and somatic mutation characteristics of the GGI. (A) Heatmap showing the expression levels of genes composing the GGI score. (B) Comparative analysis of GGI scores across different clinical and pathological characteristic groups. (C) Top 10 most frequently mutated somatic genes in High_GGI and Low_GGI groups. (D) Comparison of Tumor Mutation Burden (TMB) between High_GGI and Low_GGI groups. (E) Scatter plot illustrating the correlation between GGI and TMB. *P < .05; **P < .01; ****P < .00001. GGI = GGRGs-derived index, ns = not significant.

The GGI, composed of 5 genes, exhibits downregulated expression in the Low_GGI group and upregulated expression in the High_GGI group (Fig. 3A). The GGI demonstrates a significant correlation with patient status and stage, showing that it is significantly higher in deceased patients compared to those who are alive, and also significantly elevated in stage IV patients relative to stage II and III patients. However, no significant differences in GGI were observed among patients of different age groups or T stages (Fig. 3B). Among the top 10 most frequently mutated somatic genes, there is a distinct heterogeneity between the High_GGI group (Fig. 3C). There is no statistically significant difference in the tumor mutation burden between the High_GGI and Low_GGI groups (Fig. 3D), and no significant correlation was found between GGI and tumor mutation burden (*r* = -0.08, *P* = .48) (Fig. 3E).

### 3.4. GGI implicates molecular mechanisms underlying prognosis in UVM

**Figure 4.**
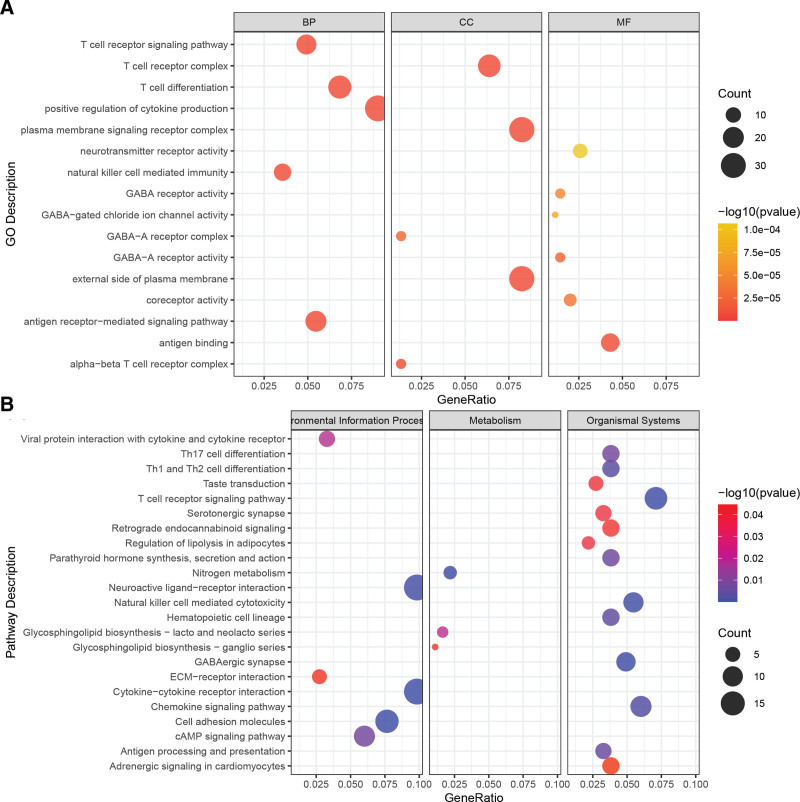
Biological processes and pathway features associated with the GGI. (A) GO enrichment analysis of differentially expressed genes between High_GGI and Low_GGI groups. (B) KEGG pathway enrichment analysis of differentially expressed genes between High_GGI and Low_GGI groups. GGI = GGRGs-derived index, GO = Gene Ontology, KEGG = Kyoto Encyclopedia of Genes and Genomes.

To elucidate the molecular underpinnings of the prognosis differences between High_GGI and Low_GGI groups, we conducted enrichment analysis on differentially expressed genes between the 2 groups. Initially, 640 differentially expressed genes were identified. Further enrichment analysis revealed that these genes are primarily enriched in immune regulation processes such as T cell differentiation and natural killer cell-mediated immunity, as well as receptor complex components (e.g., T cell receptor complex, plasma membrane signaling receptor complex), and molecular functions related to antigen binding and receptor activities (Fig. 4A). The KEGG pathway enrichment analysis demonstrated that these differentially expressed genes are significantly enriched in environmental information processing pathways including neuroactive ligand–receptor interaction and cytokine–cytokine receptor interaction, metabolism pathways like nitrogen metabolism, and organismal systems involving T cell receptor signaling pathway and chemokine signaling pathway (Fig. 4B). This suggests that the GGI may provide insights into the molecular mechanisms governing the differential prognosis observed between the High_GGI and Low_GGI groups in UVM.

### 3.5. GGI predicts drug sensitivity in UVM

**Figure 5.**
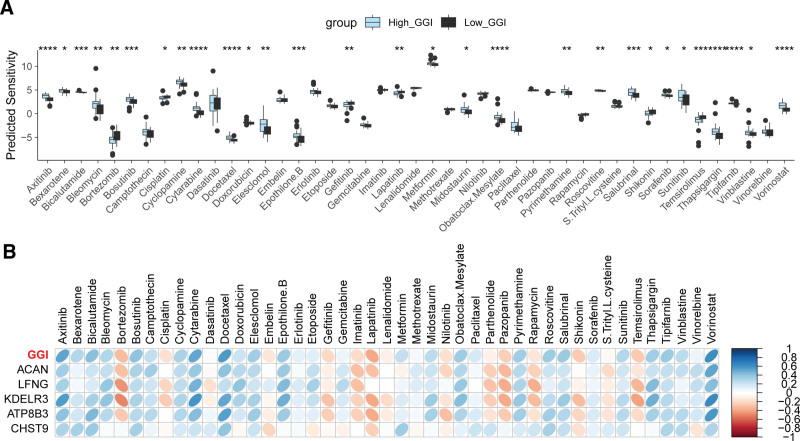
Association of the GGI with drug sensitivity. (A) Differential analysis of drug sensitivity for 45 drugs between High_GGI and Low_GGI groups. (B) Correlation analysis between GGI, its 5 constituent genes, and the sensitivity to 45 drugs. *P < .05; **P < .01; ***P < .0001; ****P < .00001. GGI = GGRGs-derived index.

Drug sensitivity analysis (Fig. 5A) revealed that patients in the High_GGI group exhibit significantly higher sensitivity to 23 drugs compared to those in the Low_GGI group, including axitinib, bexarotene, bicalutamide, bleomycin, among others. Conversely, the High_GGI group demonstrated significantly lower sensitivity to 6 drugs than the Low_GGI group, namely bortezomib, cisplatin, gefitinib, lapatinib, shikonin, and temsirolimus. The correlation between GGI expression levels and its constituent genes with drug sensitivity is depicted in Figure 5B. This figure illustrates a consistent relationship between the expression of GGI component genes and their respective drug sensitivities, aligning closely with the overall correlation pattern observed between GGI expression and drug sensitivity itself.

### 3.6. GGI correlates with the tumor microenvironment in UVM

**Figure 6.**
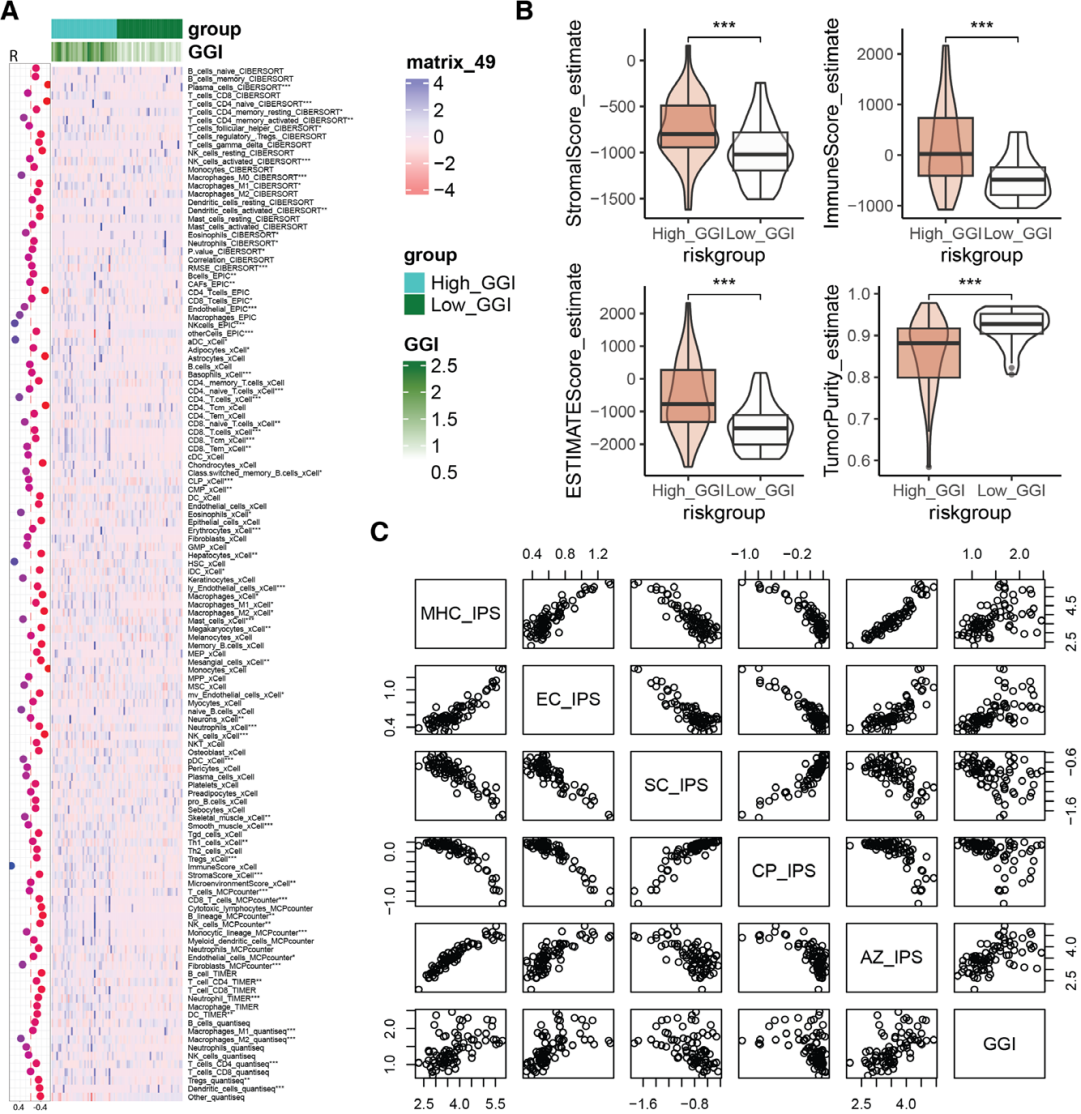
Relationship between the GGI and the tumor microenvironment. (A) Immune infiltration difference analysis and correlation analysis of GGI with immune cell infiltration between High_GGI and Low_GGI groups. *P < .05; **P < .01; ***P < .0001. (B) Differences in Stromalscore, Immunescore, ESTIMATEscore, and Tumor purity between High_GGI and Low_GGI groups. (C) Scatter plots demonstrating the correlation between MHC_IPS, EC_IPS, SC_IPS, CP_IPS, AZ_IPS, and GGI. GGI = GGRGs-derived index.

Immune infiltration analysis revealed that GGI is associated with immune cell infiltration, displaying differences between High_GGI and Low_GGI groups, including T cells, natural killer cells, neutrophils, cancer-associated fibroblasts, and macrophages (Fig. 6A). Compared to the Low_GGI group, patients in the High_GGI group exhibit higher StromalScores, ImmuneScores, and ESTIMATEScores, along with lower Tumor purity scores (Fig. 6B). Moreover, GGI shows significant positive correlations with MHC-IPS, EC_IPS, and AZ_IPS, while it negatively correlates with SC_IPS and CP_IPS (Fig. 6C). These findings indicate a relationship between GGI and immune cell infiltration as well as the tumor microenvironment.

### 3.7. Joint construction of a nomogram for UVM using GGI and age

**Figure 7.**
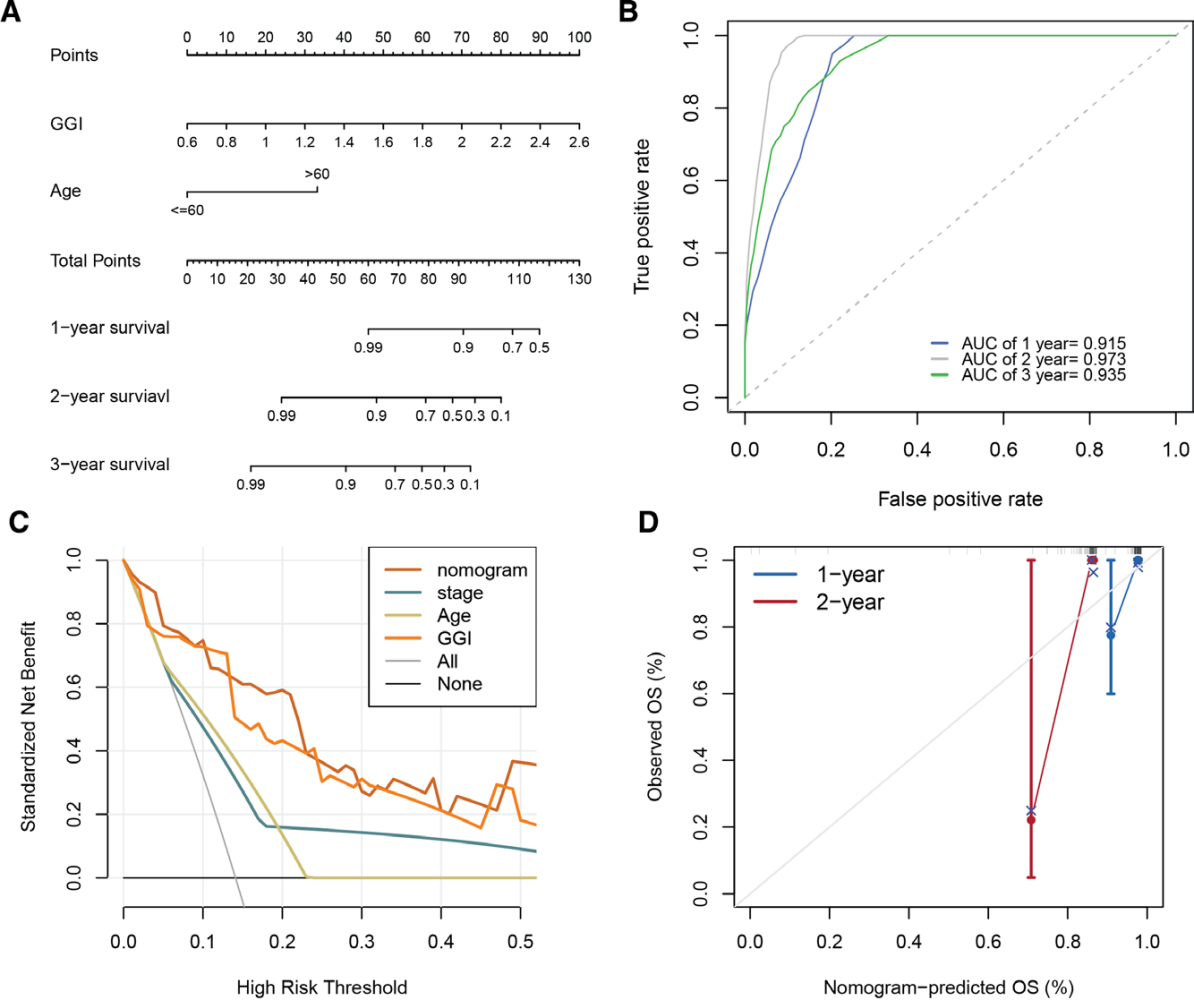
Construction of a nomogram for UVM prognosis based on the GGI. (A) Nomogram established using GGI and age as predictors. (B) ROC curve analysis demonstrating the predictive performance of the nomogram for 1-, 2-, and 3-year overall survival rates. (C) Decision curve analysis comparing the predictive utility of the nomogram against other prognostic factors for 1-year overall survival. (D) Calibration curves illustrating the agreement between the predicted and actual 1- and 2-year overall survival probabilities for the nomogram constructed with GGI and age as inputs. GGI = GGRGs-derived index, ROC = receiver operating characteristic, UVM = uveal melanoma.

Univariate Cox regression analysis identified GGI (HR [95% CI] = 21 [4.6–99], *P* = 9.10E-05), age (HR [95% CI] = 6 [1.3–28], *P* = .023), and stage (HR [95% CI] = 6.9 [1.5–31], *P* = .012) as prognostic factors for UVM. Multivariate Cox regression analysis confirmed that GGI (HR [95% CI] = 132.37 [5.36–3271.95], *P* = .003) and age (HR [95% CI] = 17.36 [1.53–196.72], *P* = .021) are independent prognostic factors for UVM (Table 1). Consequently, we constructed a nomogram for UVM prediction incorporating GGI and age (Fig. 7A), which displayed AUC values of 0.915, 0.973, and 0.935 for predicting 1-, 2-, and 3-year overall survival rates, respectively (Fig. 7B). Decision curve analysis demonstrated that this nomogram outperformed other prognostic factors (Fig. 7C). Figure 7D presents the calibration curves of the nomogram for predicting 1- and 2-year overall survival probabilities.

**Table 1:** Details of the univariate and multivariate cox regression analysis of GGI and clinical features.

Characteristics	Univariate cox				Multivariate cox			
	HR	Lower.95	Upper.95	*P*	HR	Lower.95	Upper.95	*P*
GGI	21	4.6	99	9.10E-05	132.37	5.36	3271.95	.003
Age	6	1.3	28	0.023	17.36	1.53	196.72	.021
Stage	6.9	1.5	31	0.012	9.64	0.85	110	.068
Gender	0.3	0.066	1.4	0.13	0.15	0.02	1.03	.053
T stage	4	0.84	19	0.081	2.43	0.11	53.57	.575

GGI = GGRGs-derived index.

### 3.8. Biological function of CHST9 in UVM cell

**Figure 8.**
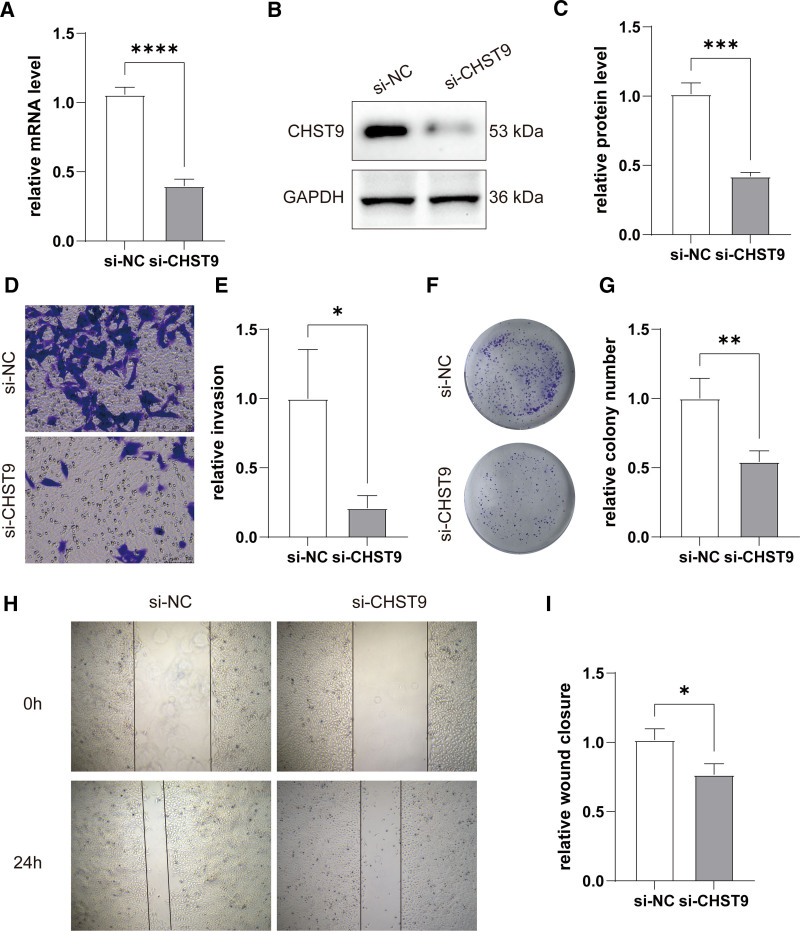
Evaluation of the biological function of CHST9 in UVM cells. (A) qPCR determination of the CHST9 mRNA level. (B) Western blot determination of the protein level of CHST9. (C) Comparison of the protein level of CHST9 between the si-NC and si-CHST9 groups. (D) Representative images of the transwell invasion assay. (E) Comparison of the relative invasive cells between the si-NC and si-CHST9 groups. (F) Representative images of the colony formation assay. (G) Comparison of the relative colony number between the si-NC and si-CHST9 groups. (H) Representative images of the wound healing assay. (I) comparison of the relative wound healing closure between the si-NC and si-CHST9 groups. *P < .05, **P < .01, ***P < .001, ****P < .0001. CHST = carbohydrate sulfotransferase protein family, UVM = uveal melanoma.

In order to assess the biological functions of CHST9 in UVM cells in the future, we conducted a CHST9 gene knockdown experiment using the OMM2.5 cell line. The results showed that the expression levels of both CHST9 mRNA and protein in the si-CHST9 group were significantly lower compared to the si-NC group (Fig. 8A–C). Transwell assays were employed to evaluate the effects of CHST9 on cell invasion in UVM cells. The number of invasive cells in the si-CHST9 group was significantly decreased compared to the control group (*P* < .05; Fig. 8D and E). Colony formation assays were carried out to further explore the effects of CHST9 inhibition on cell proliferation and colony-forming ability. As illustrated in Figure 8F and G, the number of colonies formed by OMM2.5 cells in the si-CHST9 group was significantly reduced compared to the control group (*P* < .01). Furthermore, wound-healing assays were used to measure the migratory capacity of the cells. Silencing CHST9 significantly impaired the migration ability of OMM2.5 cells, leading to a delay in wound healing (Fig. 8H and I; *P* < .05). Collectively, our findings suggest that CHST9 plays a complex role in the regulation of proliferation, metastasis, and invasiveness in UVM cells.

## 4. Discussion

The GA, an essential intracellular hub for protein and lipid synthesis, modification, and trafficking, has been demonstrated to play a critical role in key processes such as proliferation, metastasis, and drug resistance in various diseases, particularly breast cancer, thereby illuminating its association with the development and progression of malignancies. Risk signatures related to the Golgi complex have been constructed and validated across multiple cancers, including breast cancer,^[24]^ lung adenocarcinoma,^[25]^ and hepatocellular carcinoma.^[26]^ This study pioneers a systematic analysis of UVM gene expression data and clinical information to investigate the function of GGRGs in disease progression and prognosis. We identified a set of 343 GGRGs significantly associated with patient outcomes and, through consensus clustering, stratified the TCGA-UVM cohort into 2 distinct molecular subtypes that exhibit significant differences in prognosis. Moreover, principal component analysis further substantiated clear boundaries between these 2 subtypes. These findings suggest that GGRGs may serve as potential prognostic biomarkers to differentiate among different molecular subtypes of UVM and could be instrumental in predicting patients’ clinical outcomes.

Further, we constructed a GGI, which is a risk signature composed of 5 genes (LFNG, KDELR3, CHST9, ATP8B3, and ACAN) and has demonstrated excellent performance in patient prognosis assessment. The expression levels of these genes positively correlate with prognosis, suggesting their potential oncogenic functions. LFNG is an integral component of the Notch signaling pathway and is located within the trans-Golgi network. In mouse mammary gland, LFNG deletion led to deregulated Notch activation and induced basal-like and claudin-low tumors.^[27]^ Pan-cancer analysis identified LFNG as a prognostic factor across multiple cancers, including UVM, pancreatic adenocarcinoma, and low-grade gliomas.^[28]^ In prostate cancer, LFNG plays a tumor-suppressive role through differential regulation of Notch signaling.^[29]^ A previous study also identified lunatic fringe (LFNG) among 28 genes whose elevated expression predicts poor prognosis and associates with a prometastatic phenotype in mouse melanoma cells.^[30]^ KDELR3 belongs to the KDEL (Lys-Asp-Glu-Leu) receptor family, which regulates protein trafficking between the GA and endoplasmic reticulum.^[31]^ Functionally, KDELR members can bind to heterotrimeric G proteins in the Golgi complex, promoting initial protein transport.^[32]^ Consistent with prior research,^[33]^ high KDELR3 expression is indicative of poor prognosis in UVM. KDELR3, along with YWHAZ, possesses tumorigenic potential, and silencing KDELR3 suppresses tumor growth in xenograft models.^[34]^ CHST9 is a member of the carbohydrate sulfotransferase protein family (CHSTs), which are crucial components of the cytoplasmic matrix. These enzymes play a pivotal role in tissue remodeling and are implicated in cancer development.^[35,36]^ Polymorphisms in CHST9 significantly influence prognosis in early-stage triple-negative breast cancer, highlighting its clinical potential for identifying high-risk TNBC recurrence patients and guiding personalized treatment decisions.^[37]^ Our in vitro functional experiments further confirmed that inhibiting CHST9 attenuates the proliferation, migration, and invasive capabilities of UVM cells, suggesting that CHST9 may serve as a potential therapeutic target for UVM. ATP8B3 has been utilized in constructing a lung adenocarcinoma risk signature,^[38]^ and studies have shown that ATP8B3 acts as a negative regulator of cancer stemness.^[39]^ This further underscores the multifaceted roles these Golgi-related genes play in cancer progression and their potential utility as biomarkers or therapeutic targets.

A significant corollary of our study is the observed association between GGI and the infiltration levels of various immune and stromal cells, including T cells, natural killer NK cells, eosinophils, fibroblasts, macrophages, and mast cells. Independent research endeavors^[40,41]^ have substantiated that macrophages, neutrophils, mast cells, eosinophils, and activated T lymphocytes release extracellular proteases, angiogenic factors, and chemokines, which contribute to tumor progression. Moreover, the extent of immune cell infiltration has been shown to potentially influence responsiveness to cancer immunotherapy.^[42]^ Consequently, we hypothesize that dysregulation in GA-related gene mechanisms may impact the response to immunotherapy in UVM. There is a pressing need for further investigation into the potential mechanisms linking mitochondrial autophagy-related gene signatures, mitochondrial autophagy-associated risk ratios, and infiltrating immune cells, as these could provide valuable insights for personalized therapeutic strategies and pave the way for novel treatment interventions in UVM management. This line of inquiry holds promise for enhancing our understanding of the disease’s immunological landscape and tailoring targeted therapies accordingly.

Ultimately, the nomogram constructed based on 2 independent prognostic factors for UVM, namely GGI and age, furnishes a practical instrument for prognosis assessment, enabling accurate prediction of 1-, 2-, and 3-year overall survival rates in UVM patients, thereby underscoring the clinical utility of GGI in formulating personalized treatment strategies. However, our study is not without limitations. Firstly, despite the significant prognostic value of GGRGs in UVM and the development of a risk index, GGI, its predictive power and generalizability across diverse patient populations have yet to be validated in prospective studies. Secondly, while the association between GGI and UVM prognosis, as well as its relationship with immune infiltration, has been elucidated, further investigation into the precise molecular mechanisms by which GA dysfunction directly impacts the development and metastasis of UVM remains an important area for in-depth exploration. Lastly, it should be noted that the TCGA-UVM cohort predominantly consists of White individuals, with limited representation of Asian or Black populations. This underscores the critical need to incorporate data from other racial groups in future research endeavors to ensure broader applicability and generalizability of findings.

## 5. Conclusion

This study presents a novel Golgi-related signature in UVM with profound implications for prognosis prediction and tumor immune microenvironment evaluation. By examining the expression and mutations of GA-related genes in UVM samples from TCGA and GEO databases, we identified GGRGs subtypes and developed a GGI signature. Our results reveal that GGRGs play a pivotal role in UVM progression and hold potential as prognostic biomarkers. The derived GGI was associated with patient outcomes, clinical characteristics, and immune cell infiltration patterns, suggesting its utility in guiding personalized treatments. This research underscores the importance of targeting Golgi functions in developing tailored therapies to improve UVM patient survival and response rates.

## Author contributions

**Conceptualization:** Xian Ge.

**Data curation:** Xian Ge.

**Formal analysis:** Xian Ge, Fei Ge, Longqin Xiao.

**Funding acquisition:** Anting Su.

**Investigation:** Xian Ge.

**Methodology:** Xian Ge.

**Project administration:** Anting Su.

**Resources:** Xian Ge.

**Software:** Xian Ge.

**Supervision:** Anting Su.

**Validation:** Xian Ge, Fei Ge.

**Visualization:** Xian Ge, Fei Ge.

**Writing – original draft:** Xian Ge, Fei Ge.

**Writing – review & editing:** Anting Su.

## Supplementary Material


